# Familial risk of cognitive changes in the elderly

**DOI:** 10.1590/1980-5764-DN-2024-0146

**Published:** 2024-12-13

**Authors:** Johnnatas Mikael Lopes, Amanda Cirilo de Oliveira Lins, Rafael Limeira Cavalcanti, Jeisyane Acsa Santos do Nascimento, Luan Andrade Carvalho, Pedro Vitor Vieira Freire, Anna Beatriz Machado Lima, Achilles de Souza Andrade

**Affiliations:** 1 Universidade Federal do Vale São Francisco, Paulo Afonso BA, Brazil.; 2 Marinha do Brasil, Natal RN, Brazil.; 3 Universidade Federal do Rio Grande do Norte, Programa de Pós-Graduação em Fisioterapia, Natal RN, Brazil.; 4 Universidade Federal da Paraíba, João Pessoa PB, Brazil.

Dear Editor,

Normal aging involves a mild decline in some cognitive functions, while others will persist without changes. The spectrum of cognitive decline involves physiological aging, subjective cognitive decline, mild cognitive impairment (MCI), and dementia^1^. Dementia is a clinical syndrome that occurs when two or more cognitive domains are significantly compromised, with changes present in cognitive tests and considerable loss in the individual’s activities of daily living (ADL) and independence. On the contrary, MCI presents a decline in one or more cognitive domains, with changes in cognitive tests present, but without harm to their ADL, requiring minimal assistance to maintain their independence^1^.

MCI is an important risk factor for several types of dementia, and it is estimated that 10–15% of patients develop Alzheimer’s disease annually, according to a study by Petersen et al.^2^ Recognizing risk factors in patients with MCI is essential for structuring public policies focusing on secondary prevention, changing the management and prognosis of this population^2^. Given this correlation between MCI and dementia, around 50 million people worldwide have already developed some type of dementia, with an incidence of 10 million new cases each year. Estimates from the WHO^3^ indicate that in 2030 dementia is expected to affect 82 million people and 152 million in 2050. Currently, the cost of dementia reaches $1.3 trillion worldwide^4^.

Other impacts of dementia are high health costs and social and economic effects on individuals, families, and societies. This causes enormous challenges for health systems, services, and long-term support. Studies have shown that the development of these clinical conditions is due to modifiable risk factors, such as physical inactivity, smoking, alcohol consumption, and inadequate nutrition. There are also medical conditions, including hypertension, diabetes, hypercholesterolemia, depression, and obesity, that predispose the individual to MCI and other dementia diseases. The recognition of risk factors related to lifestyle, which are potentially modifiable, reveals that prevention is possible through a multisectoral approach in the public health domain, with interventions aimed at delaying MCI and preventing progression leading to some form of dementia with greater cognitive, functional, and social impairment.

According to Silva et al.^5^ the study entitled “Family Dysfunction and Cognitive Decline in Aging: The ‘Health, Wellbeing, and Aging’ (SABE) Longitudinal Population-Based Study” addresses aspects of considerable relevance for the management of cognitive decline in elderly people in clinical and public health when considering the context as a possible modulator of cognitive deterioration. However, we highlight that data analysis can be carried out differently and that this can be reflected in the results. Then, our goal here was to present this new analytical approach that can make findings more useful to the scientific community.

The outcome addressed is the presence of cognitive decline throughout the cohort follow-up period, which was measured by three psychometric instruments. As the authors’ main objective is to know the effect of family dysfunction on cognitive decline, the interesting thing is to use multivariate analysis with Poisson or Cox regression, which will produce an estimate of the effect called relative risk and hazard ratio, respectively^6^. This is necessary because logistic regression (RL) produces a biased risk estimate in prospective studies. That is, RL oversizes the confidence intervals of the odds ratio, leading to possible type II errors^6^.

The approach of presenting outcomes in terms of mean scores is not as useful, as the measure of central tendency masks changes in scores that are a considerable cognitive decline from those that are not. For example, an individual who drops 3 points and is still not considered to have cognitive decline does not have the same situation as another individual who also drops 3 points, qualifying as mild cognitive decline. It is interesting to reveal the incidence rate of decline across exposure groups in order to identify these differences.

We recommend that Poisson or Cox regression be carried out for the three cognitive outcomes in a dichotomous manner as the instruments address different cognitive dimensions, as well as building a model adjusted with the outcome of cognitive decline in the three instruments simultaneously in order to estimate the effect of the dysfunction familiar.
